# Distinguishing Relapse From Reinfection With Whole-Genome Sequencing in Recurrent Pulmonary Tuberculosis: A Retrospective Cohort Study in Beijing, China

**DOI:** 10.3389/fmicb.2021.754352

**Published:** 2021-12-08

**Authors:** Jian Du, Qing Li, Min Liu, Yufeng Wang, Zhongtan Xue, Fengmin Huo, Xuxia Zhang, Yuanyuan Shang, Shanshan Li, Hairong Huang, Yu Pang

**Affiliations:** ^1^Department of Bacteriology and Immunology, Beijing Chest Hospital, Capital Medical University/Beijing Tuberculosis and Thoracic Tumor Research Institute, Beijing, China; ^2^Provincial Center for Tuberculosis Control and Prevention, Liaoning Provincial Center for Disease Control and Prevention, Shenyang, China; ^3^Department of Laboratory Quality Control, Innovation Alliance on Tuberculosis Diagnosis and Treatment (Beijing), Beijing, China; ^4^National Clinical Laboratory on Tuberculosis, Beijing Key Laboratory on Drug-Resistant Tuberculosis Research, Beijing Chest Hospital, Capital Medical University/Beijing Tuberculosis and Thoracic Tumor Institute, Beijing, China

**Keywords:** tuberculosis, relapse, reinfection, whole-genome sequencing, pulmonary

## Abstract

**Background:** Tuberculosis recurrence is still a major problem for the control of tuberculosis, and the cause of the recurrence is still unclear.

**Methods:** We retrospectively recruited 68 pairs of samples of *Mycobacterium tuberculosis* (MTB) from recurrent TB cases in Beijing Chest Hospital between January 2008 and December 2019. The whole-genome sequencing was conducted to analyze single-nucleotide polymorphism (SNP) and to identify whether recurrent disease was due to relapse or reinfection. The BACTEC MGIT was performed to compare differences in drug susceptibility profiles between two episodes.

**Results:** 62 (91.2%) out of 68 confirmed recurrence were due to relapse, whereas the remaining six (8.8%) were due to reinfection. And there was a strong association between earlier relapse and underlying chronic diseases. In addition, the MTB isolates from non-diabetic patients had a higher mutation rate than those from diabetic patients. A community transmission was also identified in our cohort. Levofloxacin resistance was the most frequently observed drug resistance for 12.9% relapse cases.

**Conclusion:** The relapse of a previous episode in Beijing. The underlying chronic diseases are associated with an earlier TB relapse. MTB isolates were more prone to develop levofloxacin resistance than moxifloxacin resistance after FQ exposure. The patients at high-risk for relapses deserve more careful investigation.

## Introduction

Tuberculosis (TB), caused by *Mycobacterium tuberculosis* (MTB) complex, is the leading cause of death and disease from a single infectious agent and constitutes a major global public health issue (Matteelli et al., 2018; Organization, W.H, 2020a). World Health Organization guidelines recommend that patients with drug-susceptible TB should be treated with a 6-month multidrug regimen (Organization, W.H, 2020b). Although this regimen exhibits high efficacy in treatment of drug-susceptible disease, approximately 5% of these patients have a recurrence after 6months of therapy (Lambert et al., 2003). For patients whose initial disease was multidrug-resistant, recurrence of TB was particularly common (Cox et al., 2006; Xu et al., 2018). Historically, the recurrence was attributed to endogenous relapse of disease due to incapability to differentiate isolates (Witney et al., 2017). Recent advances in genotyping technologies have allowed the discrimination of relapse with the same strain that had caused previous TB episode and reinfection by a different strain (Wells et al., 2013). Inadequate treatment is the main cause for relapse (Wells et al., 2013), whereas reinfection reveals many missed TB cases circulating in the community (Zong et al., 2018). As a consequence, efforts to distinguish relapse and reinfection are of great importance to formulate effective control strategies for TB epidemics.

Since the 1980s, a series of genomic-based methods for typing MTB strains have been developed to differentiate relapse from reinfection, including IS6110 restriction fragment length polymorphism (RFLP), spoligotyping, mycobacterial interspersed repetitive units-variable number tandem repeats (MIRU-VNTR) typing, and whole-genome sequencing (WGS) (Hermans et al., 1990; Supply et al., 2006; Bryant et al., 2013). Of these available technologies, WGS provides remarkedly more information by simultaneous analysis of full genomes of multiple MTB strains, thus yielding unprecedented accuracy compared with conventional genotyping methods (Nikolayevskyy et al., 2019). Several WGS-based molecular epidemiological studies demonstrated that endogenous reactivation appeared to be the primary reason for TB recurrence in low TB-burden countries (Interrante et al., 2015), whereas reinfection may be attributed to a high proportion of TB recurrence in high TB-burden countries. These highlight the continuous interest in the geographic diversity of causes of TB recurrence of regions.

Despite great achievement in TB control and prevention, China remains one of the most high-burden countries globally. Specially, there is an unbalanced distribution of TB cases throughout the country (An et al., 2016). According to the data from nationwide survey, markedly higher incidence for TB patients is noted in the western region compared with other parts (Wang et al., 2014). In addition, the TB prevalence in rural regions is significantly higher than urban regions (Wang et al., 2014). The pre-existing disparity in TB burden has potential effect on interpretation of TB recurrences for different regions in this country. Beijing is a modernized international city in China, with an estimated TB incidence of 30.4/100,000. In spite of low incidence of TB in this municipality, more attention should be paid to patients with recurrent TB who are the source of further disease transmission in the community. However, only limited data are available on TB recurrences in our population. In order to address this concern, we retrospectively recruited 68 pairs of samples of MTB from recurrent TB cases. The WGS was conducted to identify whether recurrent disease was due to relapse or reinfection and to estimate the rate of microevolution of MTB within relapses.

## Materials and Methods

### Patients and Ethics Statement

In this retrospective, observational study, we used the BioBank of Tuberculosis System, which collected standardized data of all MTB isolates from culture-positive cases, who sought healthcare in the Beijing Chest Hospital, a national TB-designated hospital in China. Eligible patients met all of the following criteria: (i) completion of anti-TB treatment following the guidelines of National Tuberculosis Control Program; (ii) successful outcomes indicated by sputum culture conversion plus clinical findings; (iii) two or more episodes of TB which occurred in the period between January 2008 and December 2019; and (iv) a minimum time interval of 12months between two episodes (Zong et al., 2018). For patients experiencing multiple recurrences, only the first recurrent episode was included in our analysis. Data on TB cases in our cohort came from the Electronic Medical Record System in our hospital. The sociodemographic and clinical characteristics were analyzed in this study: age, sex, ethnicity, local authority of residence, TB recurrence, previous TB treatment, and site of TB disease. The study protocol was approved by the Ethics Committee of Beijing Chest Hospital, Capital Medical University and written informed consent was obtained from each participant prior to the study.

### Bacteria Subculture, DNA Purification, and Species Identification

All MTB isolates were stored in 7H9 medium supplemented with 25% glycerin at −80°C refrigerator. Prior to DNA extraction and *in vitro* drug susceptibility testing (DST), the frozen bacteria were subcultured into Löwenstein Jensen (L-J) medium. After 4weeks of incubation at 37°C, the fresh colonies were scraped from the surface of L-J medium and inactivated in a water bath at 85°C for 30min. The bacteria in the pellet were subjected to DNA extraction by using the MasterPure™ Complete DNA and RNA Purification Kit (Epicenter, Madison, WI, United States) following the manufacturer’s instructions. The quality of the extracted DNA was assessed by NanoDrop 2000c spectrophotometer (Thermo Fisher Scientific, United States). A single absorbance peak at 260nm plus a 260/280 absorbance ratio of 1.8–2.0 indicated high-quality intact DNA. In addition, the concentration was determined by Qubit 2.0 fluorometer (Invitrogen, Thermo Fisher Scientific, United States). One microliter of purified DNA was added to reaction mixture for species identification (Zeesan Biotech, Xiamen, China). A decontamination at 50°C for 2min was followed by initial denaturation at 95°C for 10min, 55cycles of denaturation at 95°C for 15s, annealing at 57°C for 20s, and plus a final extension at 78°C for 20s. The amplicons were analyzed by multicolor melting curve analysis as previously reported (Xu et al., 2019; Tan et al., 2021).

### Drug Susceptibility Testing

Because the previous DST results were produced by the absolution concentration method which was not endorsed by the World Health Organization (Organization, W.H, 2018), the fresh MTB cultures were collected for phenotypic DST with the BACTEC MGIT system (BD, Franklin Lakes, NJ). Briefly, the suspension was prepared to a turbidity equivalent to that of 0.5 McFarland standard; 0.5ml of a 1:5 dilution was used for inoculation into the MGIT containing drugs, and 0.5ml of a 1:500 dilution was used for inoculation into the drug-free control MGIT tube. After 6–14days incubation in the MGIT 960, the results were reported automatically by comparing the number of growth units (GUs) in the control tube (GU=400) and the number of GUs in the tubes containing drugs. The concentrations of drugs were as follows: isoniazid 0.1μg/ml, rifampicin 1.0μg/ml, ethambutol 5.0μg/ml, streptomycin 1.0μg/ml, levofloxacin 1.0μg/ml, moxifloxacin 0.25μg/ml, amikacin 1.0μg/ml, and capreomycin 2.5μg/ml. Isolates resistant to isoniazid and rifampicin were defined as multidrug-resistant tuberculosis (MDR-TB).

### Whole-Genome Sequencing and Sequence Analysis

Whole-genome sequencing was performed using the TIANSeq direct rapid DNA library construction kit (Tiangen, China) and the NovaSeq Illumina sequencer (Illumina, United States). After quantification and quality inspection, the extracted genomic DNA was broken into nucleic acid fragments with a length of 200–300bp by an ultrasonic interrupter (Covaris, United States) with Peak Incident Power: 175; duty factor: 10%; cycle/burst: 200; temperature: <8°C; and time: 120s. The 5′ and 3′ protruding ends of the broken DNA fragments were flattened and connected to adaptors by T4 DNA ligase. Fragment selection and purification are performed by magnetic bead purification. An amplification method was used for enrichment and magnetic bead purification was used for purification to obtain a DNA library for sequencing. Qubit Flurometer 3.0 fluorometer (Thermo Fisher Scientific, United States) and Agilent 2100 (Agilent, United States) were used to measure the concentration and length distribution of the library, respectively. The paired-end sequencing program (PE150) for the qualified library was performed on the NovaSeq Illumina sequencing platform. FastQC quality control software was used for quality control and removal of low-quality data in Raw Data, Quality filter: q, qualified_quality_phred, set the base quality value to be no less than a qualified base, the default base quality value is 15, that is, the default base quality ≥15 is qualified base, <15 is unqualified Base. u, unqualified_percent_limit, set the allowable percentage of unqualified bases, remove this read, the default is 40, that is, when the default percentage of unqualified bases >40%, remove the read; Q, disable_quality_filtering, set this parameter to disable the default quality filtering parameters (q, u). Length filter: l, length required, set the minimum length of read, the default is 15, that is, reads with length <15 are removed; L, length limit, sets the maximum length of read, the default is 0, there is no maximum length limit; and Low complexity filtering: Y, complexity threshold, set the complexity filter threshold of read, the default is 30. The MTB whole-genome sequence analysis platform (SAM-TB) software system was used to compare the sequencing data with MTB H37Rv whole-genome sequences and evaluate the sequencing depth and coverage. FastQC was used for quality control of SAM-TB, *via* adopting Cutadapt (v1.15) software to remove linkers and low-quality sequences. The retained high-quality sequence was aligned to the reference genome H37Rv (NC_000962.2) using BWA (v0.7.15). After removing duplicate reads with Picard (v2.0.1), we used SAMtools (v1.6)/VarScan (v2.3.6) to identify SNPs and short indels, and used Delly (v0.8.7) software to identify larger deletions (≥50bp). By comparison with the reference genome H37Rv, a series of information, such as single-nucleotide polymorphism (SNP) and insertion–deletion (InDel) of the target genome, were obtained and a mutation matrix of all the mutation sites of each strain was generated. SNP distance matrix, cluster strains, and genetic distance were analyzed according to the mutation matrix. SNP differences were determined between the pairs of isolates using WGS. In addition, PE/PPEs/PGRs genes, insert sequences, and other highly repetitively related positions are filtered. Reads of these regions are usually mapped to the wrong genomic coordinates, which will lead to misrecognition of SNPs. Therefore, these regions should not be considered in SNP calling. and recurrence was classified as being due to relapse if there was a difference of ≤6 SNPs (Cock et al., 2010; Jiang et al., 2011; Chen et al., 2020; Shanmugam et al., 2021). A minimum spanning tree was constructed based on pairwise whole-genome SNP differences using BioNumerics (Version5. 0, Applied Maths, Sint-Martens-Latem, Belgium) software (Mazuet et al., 2017; Rouard et al., 2019). Genotypic drug resistance prediction was predicted using the TB-Profiler (Faksri et al., 2019). The average variant depth for each isolate was ≥10 and the variant calls for drug resistance were based on the algorithms of TB-Profiler. Resistance was predicted for a total of 14 anti-TB drugs, including rifampicin, isoniazide, ethambutol, pyrazinamide, and streptomycin, and the second-line drugs Linezolid, clofazimine, bedaquiline, fluoroquinolone, capreomycin, amikacin, kanamycin, PAS, and ethionamide.

### Statistical Analysis

For the patients’ information, Epidata 3.0 software with dual-entry method was used to establish the database, and SPSS 22.0 (SPSS Inc.)^1^ statistical software was used to perform statistical analysis on the data. For continuous variables that were normally distributed, the mean±standard deviation (SD) was used; for data that were not normally distributed, the median [interquartile range; M (IQR)] was used. Counting data were described in terms of rate or composition ratio (%). Univariate and multivariate unconditional logistic regression models were used to determine the influencing factors of patients’ recurrence. Wilcoxon rank sum test was used to evaluate gene mutation rate. The Kaplan–Meier curve was generated to describe and compare the time to relapse between the different groups. *p*<0.05 was considered statistically significant.

## Results

### Description of the Study Population

**Figure 1:**
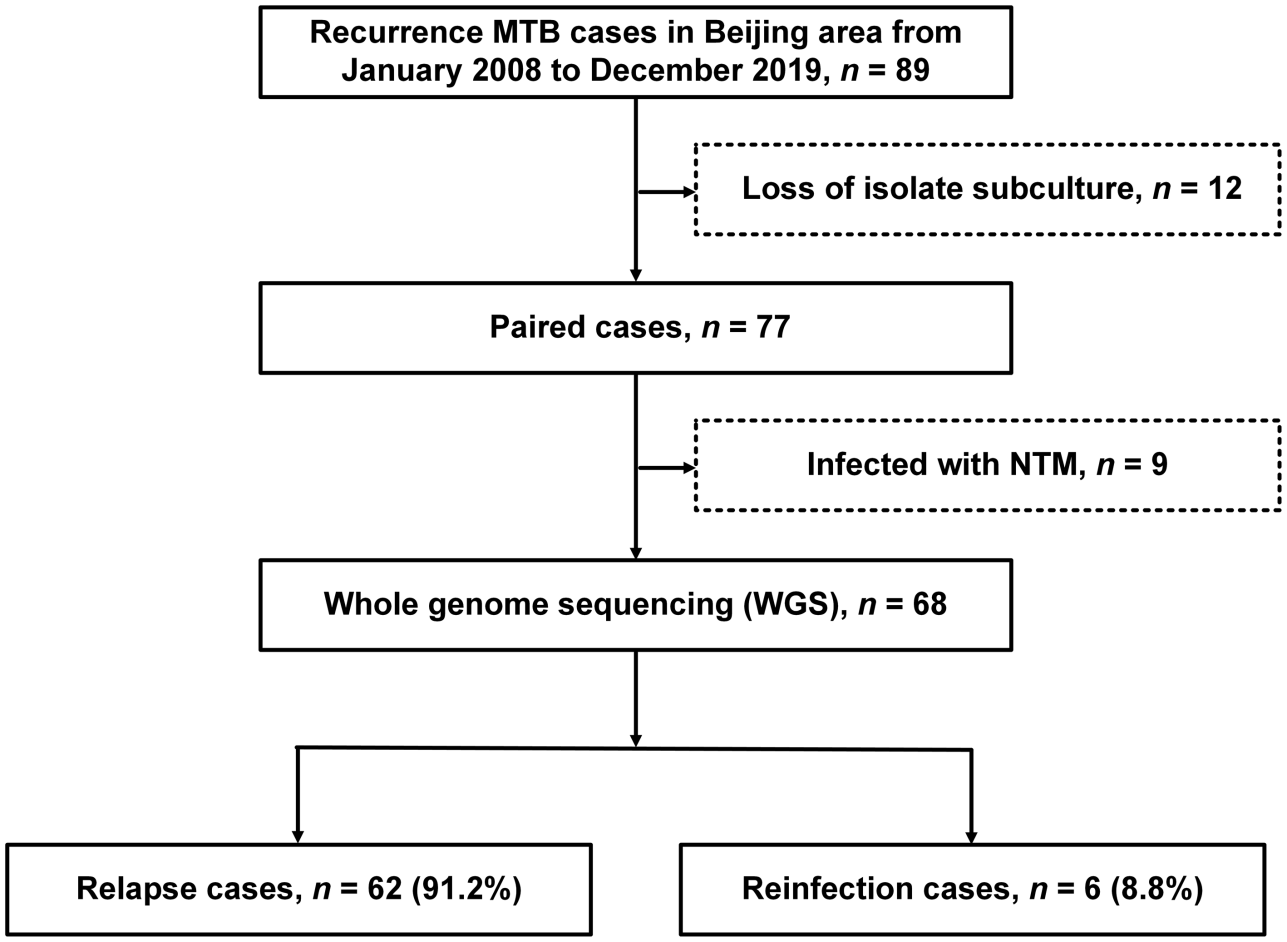
The work flow of the study. Tuberculosis (TB) patients with at least two episodes were enrolled in our study.

Between January 2008 and December 2019, a total of 89 patients meeting the inclusion criteria were included in our analysis. The majority of patients was adult men with a median age of 51years (range: 16–86years). Of the 89 subjects in the study, diabetes (*n*=29, 32.6%) was the most frequently observed comorbidity, followed by chronic disease (*n*=19, 21.4%), malnutrition (*n*=16, 18.0%), and anemia (*n*=14, 15.7%; Table 1). Figure 1 illustrates a flowchart of the enrolled samples. Among 89 recurrent TB patients, 12 (13.5%) were excluded due to subculture failure of any paired isolate. Isolates from the remaining 77 cases were analyzed by the WGS to distinguish relapse and reinfection. Of 77 cases, nine were excluded from analysis due to identification of nontuberculous mycobacteria (NTM) by WGS and multicolor melting curve analysis, leaving 68 patients with paired patients in final analysis.

**Table 1:** Clinical data of 89 patients with recurrent pulmonary tuberculosis.

Characteristics	All patients (*n*=89)
Median age (range), year	51.0 (16.0–86.0)
Male sex, *n* (%)	68 (76.4)
Cavity, *n* (%)	69 (77.5)
**Complications, *n* (%)**	
Diabetes	29 (32.6)
Malnutrition	16 (18.0)
Anemia	14 (15.7)
Autoimmune disease	4 (4.5)
Viral hepatitis	4 (4.5)
Malignant tumor	3 (3.4)
Other chronic diseases^*^	19 (21.4)

* Other chronic diseases include Cardiovascular and Cerebrovascular Diseases, Chronic Respiratory Diseases, Renal Failure, Liver Cirrhosis, excluding Diabetes and Malignant Tumors.

### Relapse and Reinfection

**Figure 2:**
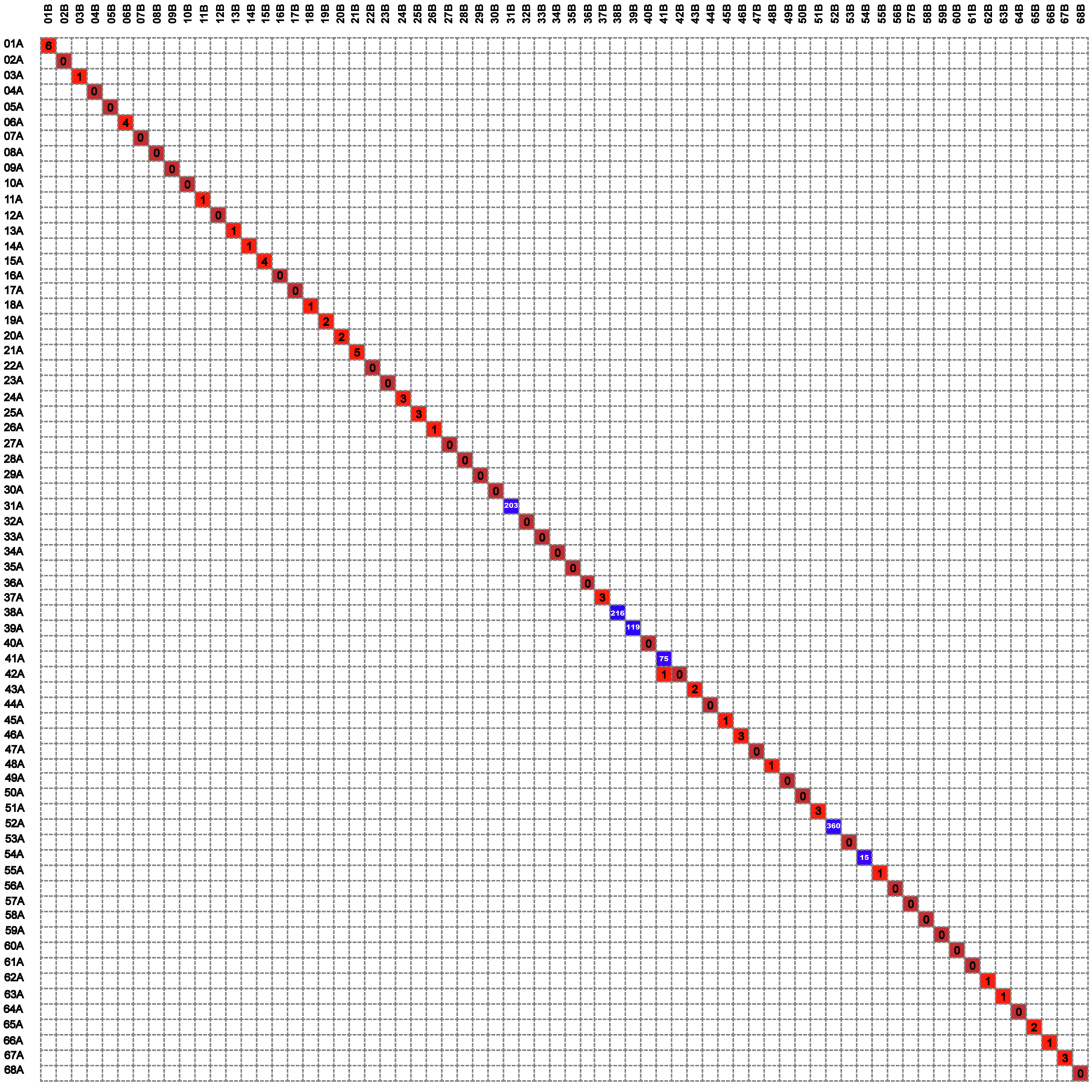
The digital matrix chart of the differential single-nucleotide polymorphisms (SNPs) of 68 patients with TB recurrence.

**Figure 3:**
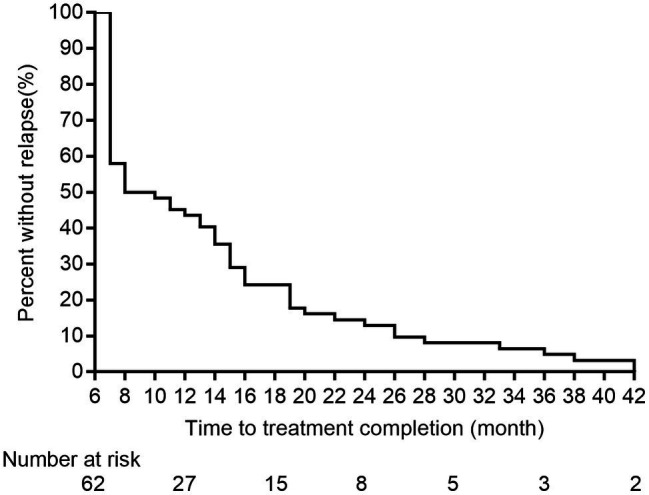
The correlation between recurrence time and recurrence ratio of the enrolled TB cases.

**Figure 4:**
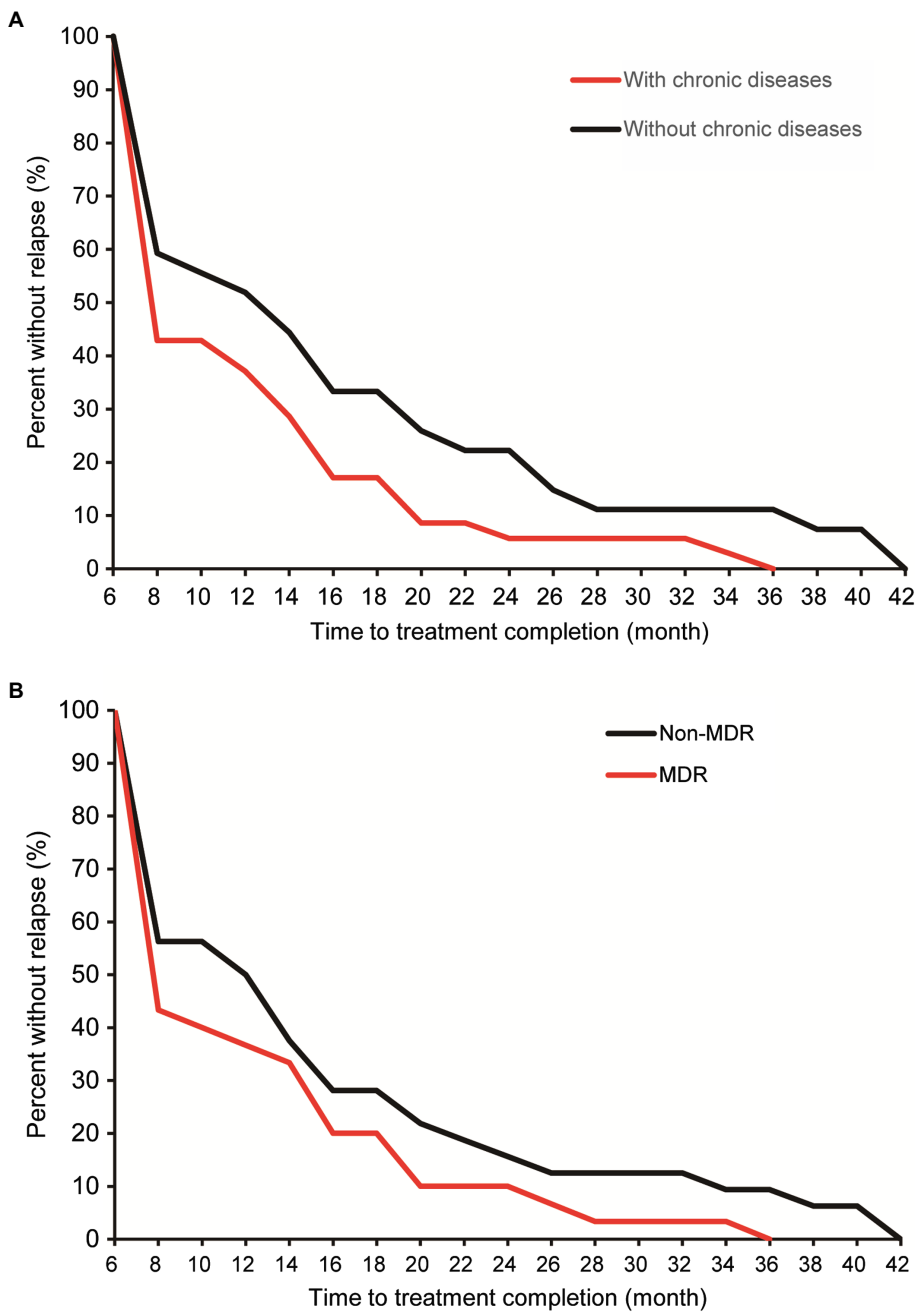
Cumulative percent of confirmed relapse tuberculosis by comorbidity **(A)** and multidrug-resistant tuberculosis (MDR-TB; **B**).

Lineage 2 was predominant among recurrent TB patients in the present study, accounting for 97.0% (132/136) of MTB strains, followed by Lineage 4 (3.0%, 4/136, Supplementary Figure S1). A comparison with SNP differences is shown in Figure 2. Two main groups were identified: 62 pairs of isolates had six or fewer SNP differences, and six pairs of samples had a dramatically higher number of SNP differences (range: 15–360). Ultimately, of the confirmed recurrence, 62 (91.2%) were due to relapse (Supplementary Table 1), whereas the remaining six (8.8%) were due to reinfection. Of 62 relapses, 35 (56.5%) occurred within 12months of treatment completion and 54 (87.1%) within 24months (Figure 3). We further assessed the time intervals to TB relapse stratified to drug susceptibility profiles and comorbidities. As shown in Figure 4, there was a strong association between earlier relapse and underlying chronic diseases (*p*=0.033). In addition, no significant differences were noted between MDR- and non-MDR-TB groups.

### Recurrence Due to Community Transmission

**Figure 5:**
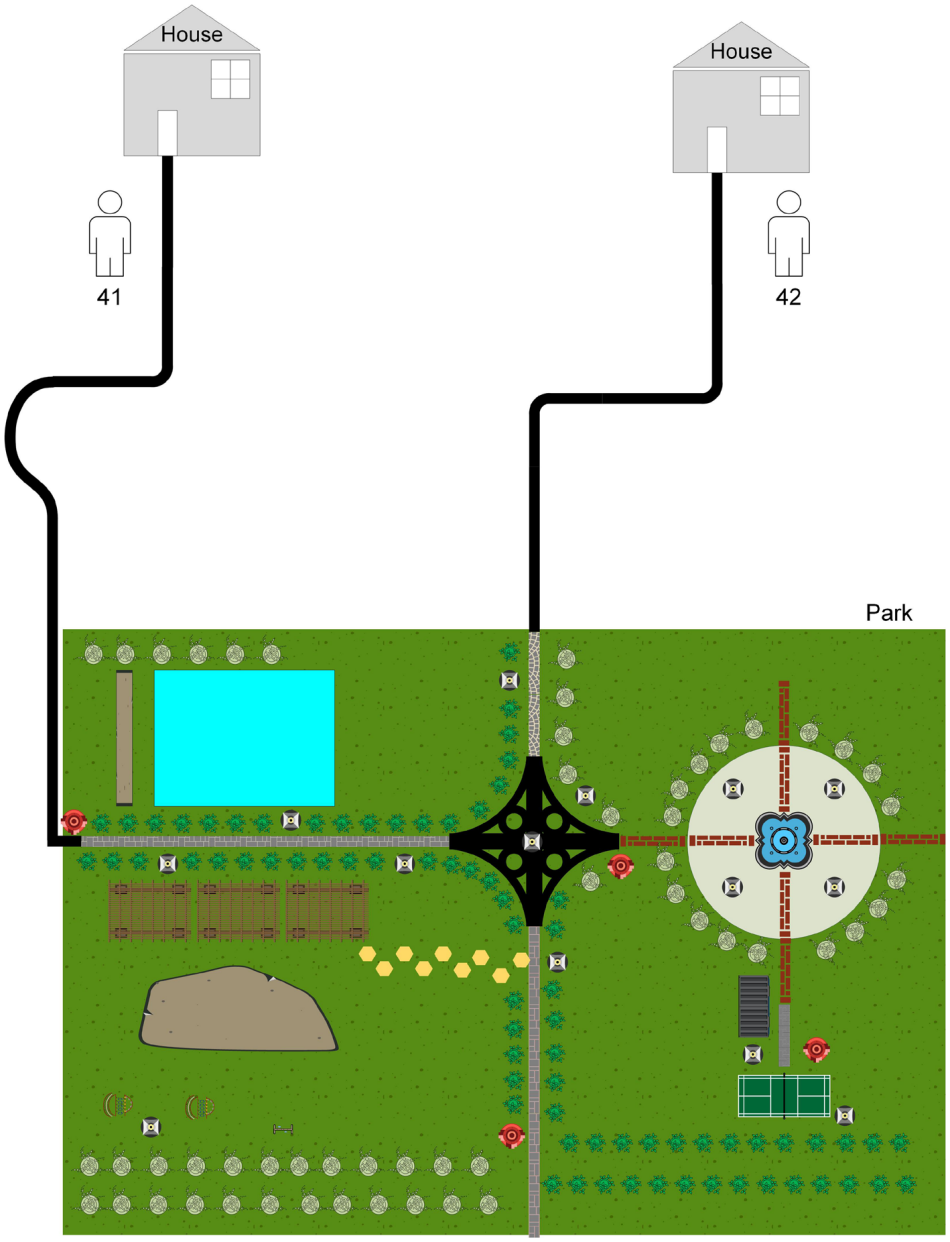
Transmission network based on epidemiological links.

Interestingly, there was one isolate that mapped closely to other patients isolates on the tree, and this merited closer attention to see if there were nosocomial infection or community transmission. As demonstrated in Figure 2, the isolate of second episode of Patient 41 showed high sequence similarity to that of Patient 42, and only one SNP was found between two isolates from different individuals. However, the two isolates of patient 41 differed by 75 SNPs, suggesting recurrence due to reinfection. We firstly reviewed their hospitalization records. Nosocomial infection was excluded since no overlapping hospitalization period occurred. Of note, both two patients lived in the neighboring blocks. Thus, we conducted interview to determine whether the community transmission could contribute to this cluster. Epidemiological data revealed that these two retired elderly individuals routinely completed morning exercises in the same park. This combined with their genotyping data supported the potential community transmission between Patient 41 and Patient 42 (Figure 5).

### Microevolution of MTB Isolates

**Figure 6:**
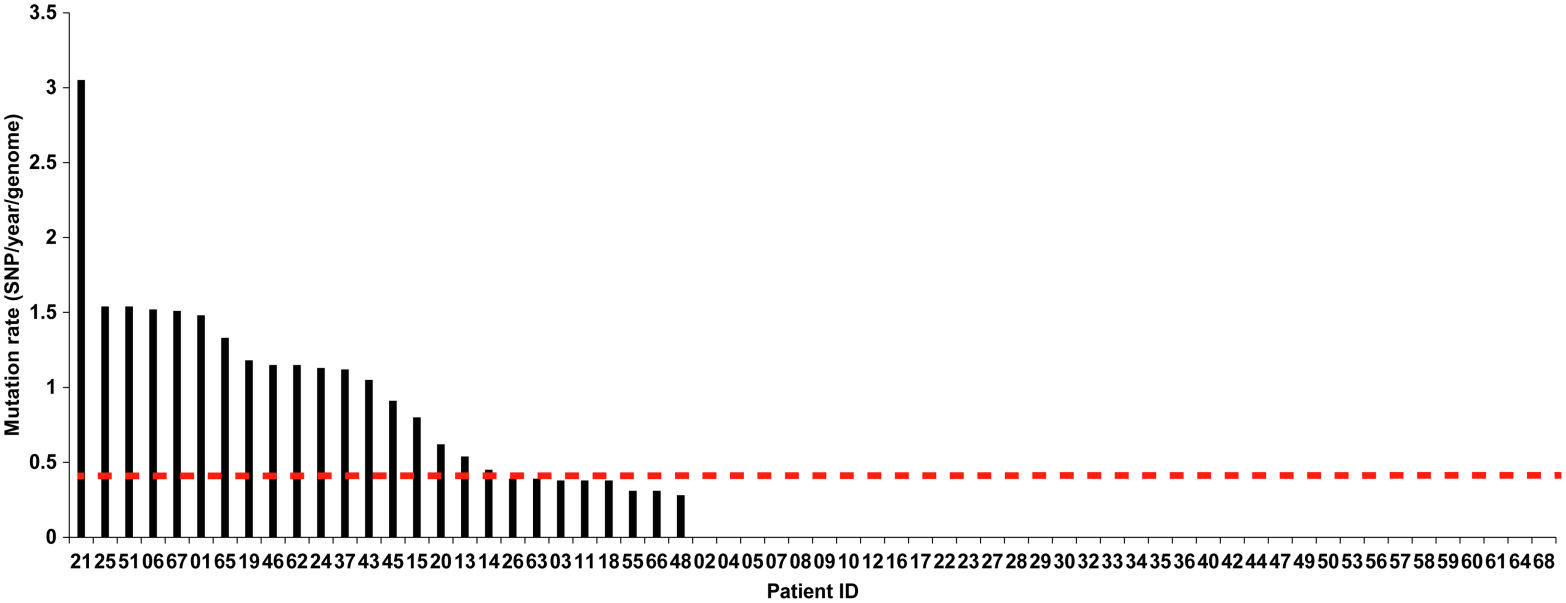
The mutation rate of the two isolates from two episodes in relapsed patients. The *X*-axis indicates the patient IDs, and the *Y*-axis indicates the mutation rate per genome per year (SNPs/year/genome).

We further analyzed the rate of microevolution of MTB from the first and second episodes of the longitudinally sampled patients (Figure 6). A rate of change in DNA sequences of 0.40 SNPs per genome per year [95% confidential interval (CI) 0.24–0.56] was noted in our study. We recorded a significant higher mutation rate in non-diabetic patients (0.53 SNPs per genome per year) compared with that in diabetic patients (0.14 SNPs per genome per year, *p* =0.012). By contrast, no difference was observed in mutation rate between MDR-TB and non-MDR-TB patients (0.39 SNPs per genome per year for MDR-TB vs. 0.42 SNPs per genome per year for non-MDR-TB, *p* =0.919).

### Emergence of Drug Resistance After Relapse

**Figure 7:**
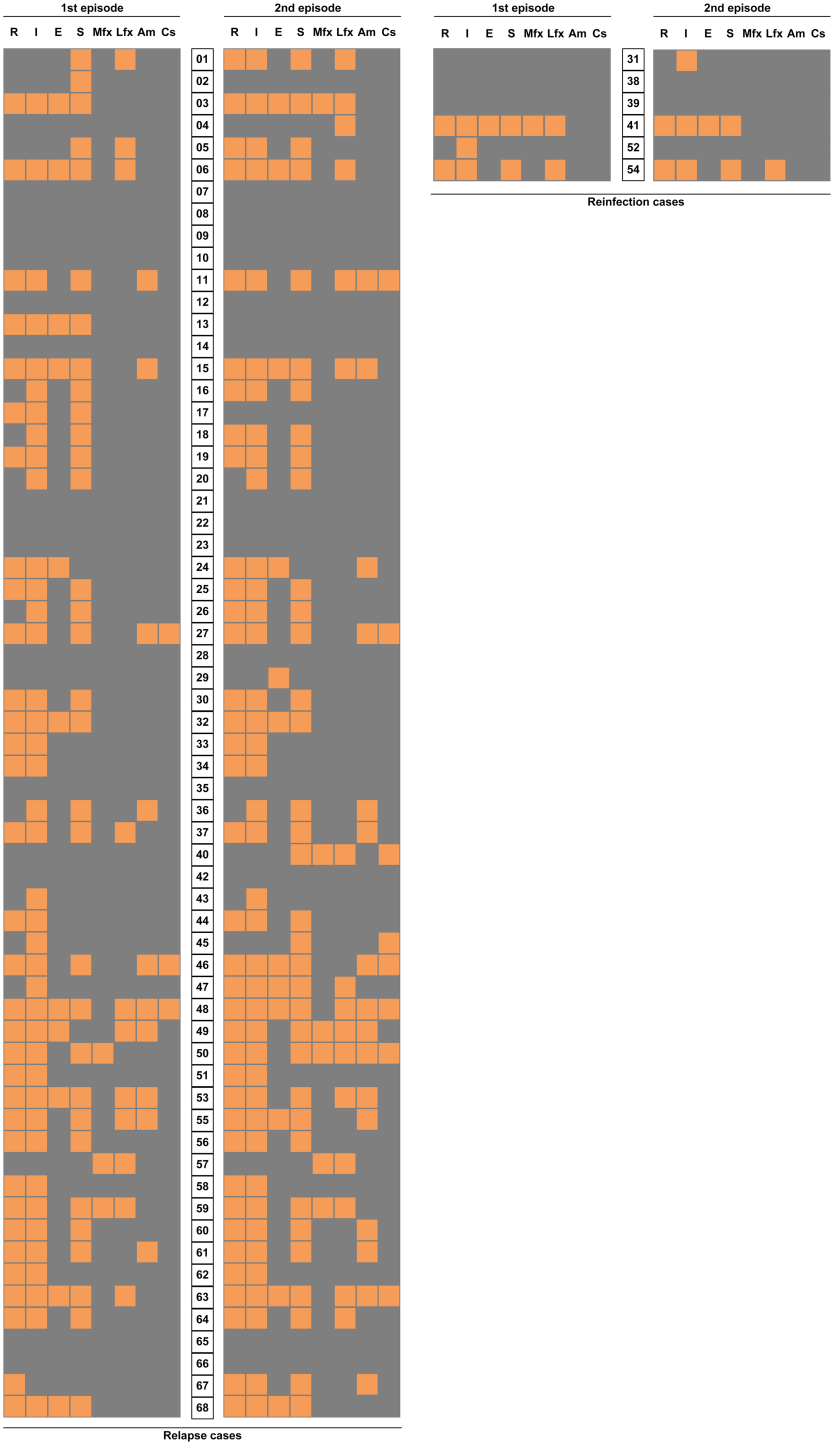
Comparison of drug sensitivity distribution between the two TB episodes. Each grid represents the result of drug susceptibility test with isolates from two episodes of each patient. The results are shown in an orange-gray color scale, where orange represents resistance to the corresponding drug and gray represents susceptivity to the corresponding drug. R, rifampicin; I, isoniazid; E, ethambutol; S, streptomycin; Mfx, moxifloxacin; Lfx, levofloxacin; Am, amikacin; and Cs, capreomycin.

We further analyzed the differences in drug susceptibility profiles between first and second TB episode. As shown in Figure 7, levofloxacin resistance was the most frequently observed for 8 (12.9%) of 62 relapse cases, followed by rifampicin (9.7%, *n* =6), streptomycin (9.7%, *n* =6), and amikacin (9.7%, *n* =6). For moxifloxacin, another fluoroquinolone (FQ), 2 (3.2%) paired isolates exhibited changes from sensitivity to resistance. Statistical analysis revealed that this proportion was significantly lower than that for levofloxacin (*p* =0.048), indicating that MTB isolates were more prone to develop levofloxacin resistance than moxifloxacin resistance after FQ exposure. A deep analysis was conducted to compare the drug susceptibility profiles between two episodes by WGS. As shown in Supplementary Table 2, FQ resistance was recorded in 22.6% (14/62) of relapse cases, followed by kanamycin (11.3%, 7/62), and pyrazinamide (8.1%, 5/62). In contrast, no bedaquiline and linezolid resistance were noted in these relapse cases.

## Discussion

To our knowledge, this is one of the largest studies investigating the recurrence of TB with WGS in China. The current study confirmed that the recurrence was majorly due to the relapse of a previous episode and that exogenous reinfection approximately accounted for 9% of recurrent cases. The extent to which exogenous reinfection occurs depends on the prevalence of pulmonary TB, and the greater likelihood of exogenous reinfection is associated with higher prevalence of this diseases (Bandera et al., 2001). In low-incidence countries, researchers have found the percentage of reinfection ranging from 4% in the United States and Canada to 23% in Italy (Jasmer et al., 2004; Schiroli et al., 2015). By contrast, reinfection was the major reason for the recurrent cases, ranging from 17% in Georgia to 69% in South Africa (Charalambous et al., 2008; Maghradze et al., 2019). In studies of MTB isolates circulating in China, the rate of reinfection among pulmonary TB patients exhibited great diversity, ranging from 47% in Jiangsu to 61% in Shanghai (Shen et al., 2006; Shao et al., 2021). This significant differences may be partially explained by the varying TB incidences across geographic regions and time. In addition, a molecular epidemiological study by Shen et al. (2017) revealed 42% of recurrent cases had different genotypes, indicating that the immunity conferred by initial infection is of importance to protect the individuals from reinfection by the same genotypes. Therefore, we speculate that the predominant prevalence of Beijing genotype serves as another potential contributor to low proportion of reinfection in our observation.

Another interesting finding of our study was a community transmission of pulmonary TB *via* WGS-based clustering was identified. The neighboring blocks of residence of two patients within this cluster attracted our attention. Further epidemiological survey revealed that there was temporal-spatial overlap between Patient 41 and Patient 42 during morning exercises. On the one hand, our results underline the great importance of monitoring the recurrence to timely recognize index cases and block transmission. A cost-effectiveness concern is raised how long we should follow up to monitor the recurrence. In consistent with previous findings, approximate 56.5% of relapses occurred within 12months of treatment completion. Therefore, the patients at high-risk for relapses deserve more careful investigation. On the other hand, exogenous reinfection in Patient 41 emphasizes the host susceptibility to tubercle bacilli. Further experimental studies are required to elucidate the molecular mechanisms in susceptible populations.

The emergence of drug resistance in MTB after relapse undermines the efficacy of subsequent anti-TB treatment. In this study, we noted that levofloxacin resistance is the most frequently observed drug resistance among second episodes. Several potential explanations could be responsible for the high levofloxacin resistance rate. First, empirical treatment with a fluoroquinolone is always provided for patients with suspected pulmonary infection due to its potent activity against pathogens in China (Zhang et al., 2014). Therefore, the abuse and misuse of FQs may play an important role in the accumulation of drug-resistant MTB in this country. In line with our findings, numerous previous reports have demonstrated dramatically increased prevalence of FQ-resistant MTB during the past decades in China (Pang et al., 2017; Huo et al., 2020). Second, previous experimental evidence showed that FQs are effective inducers of the SOS DNA repair system (Schröder et al., 2013). Consequently, the boosted hydrogen peroxide production increases the spontaneous mutation rate of tubercle bacilli, thereby resulting in the emergence of FQ resistance. Taken together, more actions are required to minimize inappropriate usage of FQs in clinical practice, which is essential for control of antibiotic-resistant bacteria.

Findings in a primate model with active and latent TB define a molecular clock rate of ∼0.3–0.5 SNPs/year (Ford et al., 2011), which are consistent with the estimated 0.3–0.5 SNP changes per year per genome from epidemiological studies (Pérez-Lago et al., 2014; Guerra-Assunção et al., 2015a). Based on our data, a slightly higher rate (~0.5 SNPs/year) was noted in our relapse cohort. We propose two possible explanations for the diversity. First, MTB isolates from linage 2 (East Asian lineage and Beijing sublineage) had higher mutation rate than isolates from lineage 4, accounting for the optimal adaptability and an emergence ratio of drug resistance (Guerra-Assunção et al., 2015b). The predominance of Beijing genotype in our study may be associated with higher mutation rate among relapse cases. Alternatively, a recent study by Colangeli et al. (2020) demonstrated a significant difference in mutation rate between two phases of latent infection in humans, compromising of early latency with high mutation rate and late latency with slow mutation rate. Given the fact that 87.1% of patients in our study relapsed within 2years, the tubercle bacilli were more properly be considered an incubation period of early latency, thus resulting in a higher mutation rate.

Our analysis suggested that the dormant tubercle bacilli in non-diabetic patients harbored a higher mutation rate than diabetic patients. Recently, Liu et al. identified elevated bacterial mutation rates in MTB isolates from HIV-negative but not HIV-positive individuals, indicating that the *in vivo* molecular clock for MTB was modulated by host immune (Liu et al., 2020). This phenomenon is consistent with the growing knowledge of impaired immunity in diabetic hosts, who produce decreased ROS stress compared with non-diabetic hosts (Newsholme et al., 2007; Rendra et al., 2019). This heterogeneity perhaps helps explain why isolates from non-diabetic hosts accumulate higher numbers of SNPs between the first and second episode.

We also acknowledged several obvious limitations to our study. First, despite the enrollment of all paired isolates from relapses, the relative sample size weakened the significance of our conclusion. Second, due to the predominance of relapse in our cohort, it is difficult to analyze the risk factors associated with relapse and reinfections. Third, crude mutation rates of MTB isolates were estimated in the present study using the mixed population of tubercle bacilli rather than isolation of single colonies. Therefore, this mutation rate may be underestimated. Fourth, we observed that some samples showed loss of antibiotic resistance in the second episode. In addition to the viability of the bacteria, the potential laboratory errors may be an explanation for this phenomenon despite performing quality control on each batch. Finally, the short posttreatment relapse interval may lead to the underestimation of the prevalence of reinfection among our recurrent patients considering that MTB is characterized by a low mutation rate (Eldholm and Balloux, 2016). Nevertheless, our study provides new insights to help formulate effective strategies against TB recurrence in a Chinese metropolis city with low TB incidence.

## Conclusion

Our data demonstrate that the recurrence is majorly due to the relapse of a previous episode in Beijing. The underlying chronic diseases are associated with an earlier TB relapse. Levofloxacin resistance is the most frequently observed drug resistance among second episodes. In addition, the dormant tubercle bacilli in non-diabetic patients harbored a higher mutation rate than diabetic patients. An identified community transmission highlights that the patients at high-risk for relapses deserve more careful investigation.

## Data Availability Statement

The datasets presented in this study can be found in online repositories. The names of the repository/repositories and accession number(s) can be found in the article/Supplementary Material.

## Ethics Statement

The studies involving human participants were reviewed and approved by the Ethics Committees of the Beijing Chest Hospital, Capital Medical University. The patients/participants provided their written informed consent to participate in this study.

## Author Contributions

YP, HH, SL, and JD: conceptualization. QL, ZX, and JD: data curation. QL and ML: formal analysis. ML, YW, ZX, FH, XZ, and YS: methodology. YP and JD: project administration. JD, QL, YW, and ZX: writing - original draft. YP, JD, HH, ML, and SL: writing - review and editing. All authors contributed to the article and approved the submitted version.

## Funding

This study was supported by the Capital’s Funds for Health Improvement and Research (2020-1-1041), the National Key Project (2018ZX10722301), the Beijing Hospitals Authority Ascent Plan (DFL20191601), and the Beijing Hospitals Authority Clinical Medicine Development of Special Funding (ZYLX202122).

## Conflict of Interest

The authors declare that the research was conducted in the absence of any commercial or financial relationships that could be construed as a potential conflict of interest.

## Publisher’s Note

All claims expressed in this article are solely those of the authors and do not necessarily represent those of their affiliated organizations, or those of the publisher, the editors and the reviewers. Any product that may be evaluated in this article, or claim that may be made by its manufacturer, is not guaranteed or endorsed by the publisher.

## Footnotes

^1^ http://www.SPSS.com.hk
