# Supplementary material for: Distinguishing Relapse From Reinfection With Whole-Genome Sequencing in Recurrent Pulmonary Tuberculosis: A Retrospective Cohort Study in Beijing, China
Source: Front Microbiol. 2021 Dec 8;12:754352. doi: 10.3389/fmicb.2021.754352 (PMC8693897; doi:10.3389/fmicb.2021.754352)

Supplementary Material

# Supplementary Tables

Supplemental Table 1 Differential SNP in 62 patients with endogenous relapse

| **Patient ID** | **Position** | **Ref ：alt*** | **Type** | **Label** | **Codon** | **Gene name** | **Function** | **Class** |
| --- | --- | --- | --- | --- | --- | --- | --- | --- |
| 1 | 162894 | G:T | Nonsynonymous | Rv0135c | TCG-TAG | Rv0135c | Possible transcriptional regulatory protein | Regulatory proteins |
|  | 1600451 | T:C | Nonsynonymous | Rv1425 | TTG-TCG | Rv1425 | Possible triacylglycerol synthase | Lipid metabolism |
|  | 2155213 | T:C | Nonsynonymous | Rv1908c | TGG-TCG | *katG* | Catalase-peroxidase-peroxynitritase T KatG | Virulence and adaptation |
|  | 3704461 | A:G | Nonsynonymous | Rv3317 | AGC-GGC | *sdhD* | Probable succinate dehydrogenase SdhD | Intermediary metabolism and respiration |
|  | 3726273 | C:T | synonymous | Rv3340 | GAC-GAT | *metC* | Probable O-acetylhomoserine sulfhydrylase MetC | Intermediary metabolism and respiration |
|  | 3979679 | C:A | synonymous | Rv3540c | GGG-GGT | *ltp2* | Probable lipid transfer protein or keto acyl-CoA thiolase Ltp2 | Lipid metabolism |
| 3 | 2528012 | C:A | Nonsynonymous | Rv2253 | CCC-CAC | Rv2253 | Possible secreted unknown protein | Cell wall and processes |
| 6 | 1627994 | C:G | synonymous | Rv1448c | CGG-CGC | *tal* | Probable transaldolase Tal | Intermediary metabolism and respiration |
|  | 1963040 | T:C | Nonsynonymous | Rv1736c | AGC-GGC | *narX* | Probable nitrate reductase NarX | Intermediary metabolism and respiration |
|  | 2569846 | A:C | synonymous | Rv2298 | GCA-GCC | Rv2298 | Conserved protein | Conserved hypotheticals |
|  | 2819576 | A:G | Nonsynonymous | Rv2504c | TCG-CCG | *scoA* | Probable succinyl-CoA:3-ketoacid-coenzyme A transferase | Lipid metabolism |
| 11 | 784189 | A:G | Nonsynonymous | Rv0684 | AAC-GAC | *fusA1* | Probable elongation factor G FusA1 | Information pathways |
| 13 | 555862 | A:G | Nonsynonymous | Rv0465c | TAC-CAC | Rv0465c | Possible transcriptional regulatory protein | Regulatory proteins |
| 14 | 2851166 | T:A | Nonsynonymous | Rv2526 | TTG-ATG | *vapB17* | Possible antitoxin VapB17 | Virulence and adaptation |
| 15 | 779095 | G:A | Nonsynonymous | Rv0678 | GCG-ACG | Rv0678 | Conserved protein | Conserved hypotheticals |
|  | 1674723 | C:G | Nonsynonymous | Rv1484 | TTC-TTG | *inhA* | NADH-dependent enoyl-[acyl-carrier-protein] reductase InhA | Lipid metabolism |
|  | 1870392 | C:T | synonymous | Rv1656 | GGC-GGT | *argF* | Probable ornithine carbamoyltransferase, anabolic ArgF | Intermediary metabolism and respiration |
|  | 2102875 | G:C | Nonsynonymous | Rv1854c | TAC-TAG | *ndh* | Probable NADH dehydrogenase Ndh | Intermediary metabolism and respiration |
| 18 | 158883 | T:C | Nonsynonymous | Rv0131c | CAA-CGA | *fadE1* | Probable acyl-CoA dehydrogenase FadE1 | Lipid metabolism |
| 19 | 186529 | T:C | Nonsynonymous | Rv0157A | GAC-GGC | Rv0157A | Conserved protein | Conserved hypotheticals |
|  | 2079190 | T:A | synonymous | Rv1833c | GTA-GTT | Rv1833c | Possible haloalkane dehalogenase | Intermediary metabolism and respiration |
| 20 | 1794462 | C:T | synonymous | Rv1593c | CAG-CAA | Rv1593c | Conserved protein | Conserved hypotheticals |
|  | 2019512 | C:T | Nonsynonymous | Rv1783 | CGC-TGC | *eccC5* | ESX-5 type VII secretion system protein | Cell wall and processes |
| 21 | 1474095 | A:C | Small RNA | MTB000020 | A-C | *rrl* | Ribosomal RNA 23S | Stable RNAs |
|  | 2895367 | C:T | synonymous | Rv2571c | GTG-GTA | Rv2571c | Probable transmembrane alanine and valine and leucine rich protein | Cell wall and processes |
|  | 4017591 | A:T | Nonsynonymous | Rv3575c | CTG-CAG | Rv3575c | Transcriptional regulatory protein (probably LacI-family) | Regulatory proteins |
|  | 4017594 | C:T | Nonsynonymous | Rv3575c | GGC-GAC | Rv3575c | Transcriptional regulatory protein (probably LacI-family) | Regulatory proteins |
|  | 4017598 | G:C | Nonsynonymous | Rv3575c | CTC-GTC | Rv3575c | Transcriptional regulatory protein (probably LacI-family) | Regulatory proteins |
| 24 | 1768685 | A:C | Nonsynonymous | Rv1563c | TAC-GAC | *treY* | Maltooligosyltrehalose synthase TreY | Virulence and adaptation |
|  | 2247694 | T:C | Nonsynonymous | Rv2002 | GTC-GCC | *fabG3* | Possible 20-β-hydroxysteroid dehydrogenase FabG3 | Virulence and adaptation |
|  | 4327466 | T:C | Nonsynonymous | Rv3854c | GAG-GGG | *ethA* | Monooxygenase EthA | Lipid metabolism |
| 25 | 1356752 | A:C | Nonsynonymous | Rv1213 | AAT-ACT | *glgC* | Glucose-1-phosphate adenylyltransferase GlgC | Intermediary metabolism and respiration |
|  | 1651959 | G:A | Nonsynonymous | Rv1464 | GCC-ACC | *csd* | Probable cysteine desulfurase Csd | Intermediary metabolism and respiration |
|  | 3036498 | A:G | Nonsynonymous | Rv2724c | CTG-CCG | *fadE20* | Probable acyl-CoA dehydrogenase FadE20 | Lipid metabolism |
| 26 | 764817 | T:G | Nonsynonymous | Rv0668 | GTG-GGG | *rpoC* | DNA-directed RNA polymerase (β' chain) RpoC | Information pathways |
| 37 | 1127774 | A:G | Nonsynonymous | Rv1008 | TAT-TGT | *tatD* | Probable deoxyribonuclease TatD (YJJV protein) | Cell wall and processes |
|  | 1647276T | C:T | synonymous | Rv1461 | TTC-TTT | Rv1461 | Conserved protein | Conserved hypotheticals |
|  | 2819408 | C:T | Nonsynonymous | Rv2504c | GAT-AAT | *scoA* | Probable succinyl-CoA:3-ketoacid-coenzyme A transferase (α subunit) ScoA | Lipid metabolism |
| 43 | 1399802 | T:C | Nonsynonymous | Rv1252c | ACG-GCG | *lprE* | Probable lipoprotein LprE | Cell wall and processes |
|  | 1902230 | C:T | synonymous | Rv1677 | GAC-GAT | *dsbF* | Probable conserved lipoprotein DsbF | Cell wall and processes |
| 45 | 555873 | T:G | Nonsynonymous | Rv0465c | GAG-GCG | Rv0465c | Possible transcriptional regulatory protein | Regulatory proteins |
| 46 | 2562973 | C:T | synonymous | Rv2290 | TAC-TAT | *lppO* | Probable conserved lipoprotein LppO | Cell wall and processes |
|  | 2851166 | T:A | Nonsynonymous | Rv2526 | TTG-ATG | *vapB17* | Possible antitoxin VapB17 | Virulence and adaptation |
|  | 2967905 | C:T | synonymous | Rv2643 | CCC-CCT | *arsC* | Probable arsenic-transport integral membrane protein ArsC | Cell wall and processes |
| 48 | 1337474 | A:G | Nonsynonymous | Rv1194c | CTG-CCG | Rv1194c | Conserved protein | Conserved hypotheticals |
| 51 | 3042438 | C:T | Nonsynonymous | Rv2729c | GGC-GAC | Rv2729c | Probable conserved integral membrane alanine valine and leucine rich protein | Cell wall and processes |
|  | 3336738 | A:T | Delete | Rv2980-Rv2981c | del_A | Rv2980-ddlA | Possible conserved secreted protein or Probable D-alanine-D-alanine ligase DdlA | Cell wall and processes |
|  | 3595772 | A:G | synonymous | Rv3219 | GTA-GTG | *whiB1* | Transcriptional regulatory protein WhiB-like WhiB1 | Regulatory proteins |
| 55 | 3689763 | A:G | synonymous | Rv3303c | GCT-GCC | *lpdA* | NAD(P)H quinone reductase LpdA | Intermediary metabolism and respiration |
| 62 | 1228636 | C:T | Nonsynonymous | Rv1099c | GAC-AAC | *glpX* | Fructose 1,6-bisphosphatase GlpX | Intermediary metabolism and respiration |
| 63 | 779383 | C:T | Nonsynonymous | Rv0678 | CGA-TGA | Rv0678 | Conserved protein | Conserved hypotheticals |
| 65 | 2539569 | T:C | synonymous | Rv2265 | GAT-GAC | Rv2265 | Possible conserved integral membrane protein | Cell wall and processes |
|  | 3662187 | C:T | synonymous | Rv3280 | CCC-CCT | *accD5* | Probable propionyl-CoA carboxylase beta chain 5 AccD5 | Lipid metabolism |
| 66 | 2851165 | A:G | synonymous | Rv2526 | ACA-ACG | *vapB17* | Possible antitoxin VapB17 | Virulence and adaptation |
| 67 | 656414 | G:C | Nonsynonymous | Rv0565c | CTT-GTT | Rv0565c | Probable monooxygenase | Intermediary metabolism and respiration |
|  | 2243009 | G:T | Nonsynonymous | Rv1998c | TCC-TAC | Rv1998c | Conserved protein | Conserved hypotheticals |
|  | 3448637 | G:T | Nonsynonymous | Rv3083 | TGG-TTG | Rv3083 | Probable monooxygenase (hydroxylase) | Intermediary metabolism and respiration |

* ref : alt reference : alteration

Supplemental Table 2 Differences in drug susceptibility profiles between the first and second episode by genotypic drug susceptibility testing.

|  | Relapse n=62(%) | | | Reinfection n=6(%) | | |
| --- | --- | --- | --- | --- | --- | --- |
|  | S→R | R→S | No change | S→R | R→S | No change |
| Rifampicin | 3(4.8) | 0(0) | 59(95.2) | 1(16.7) | 1(16.7) | 4(66.7) |
| Isoniazid | 2(3.2) | 0(0) | 60(96.8) | 1(16.7) | 1(16.7) | 4(66.7) |
| Ethambutol | 4(6.5) | 0(0) | 58(93.5) | 0(0) | 1(16.7) | 5(83.3) |
| Pyrazinamide | 5(8.1) | 0(0) | 57(91.9) | 0(0) | 1(16.7) | 5(83.3) |
| Streptomycin | 3(4.8) | 0(0) | 59(95.2) | 0(0) | 1(16.7) | 5(83.3) |
| Linezolid | 0(0) | 0(0) | 62(100) | 0(0) | 0(0) | 6(100) |
| Clofazimine | 0(0) | 0(0) | 62(100) | 0(0) | 0(0) | 6(100) |
| Bedaquiline | 0(0) | 0(0) | 62(100) | 0(0) | 0(0) | 6(100) |
| Fluoroquinolone | 14(22.6) | 0(0) | 48(77.4) | 0(0) | 1(16.7) | 5(83.3) |
| Capreomycin | 7(11.3) | 0(0) | 55(88.7) | 0(0) | 0(0) | 6(100) |
| Amikacin | 6(9.7) | 0(0) | 56(90.3) | 0(0) | 0(0) | 6(100) |
| Kanamycin | 7(11.3) | 0(0) | 55(88.7) | 0(0) | 0(0) | 6(100) |
| Para-aminosalicylic acid | 3(4.8) | 0(0) | 59(95.2) | 1(16.7) | 0(0) | 5(83.3) |
| Ethionamide | 0(0) | 1(1.6) | 61(98.4) | 0(0) | 0(0) | 6(100) |
| Total | 26(41.9) | 1(1.6) | 35(56.5) | 3(50) | 1(16.7) | 2(33.3) |

Table S3 The correspondence between Strain ID and the Run Accession Number.

| patient ID | Strain ID | Project ID | Sample ID | Experiment ID | Run ID |
| --- | --- | --- | --- | --- | --- |
| 01 | 1st strain | CNP0001828 | CNS0390036 | CNX0330451 | CNR0403245 |
|  | 2nd strain | CNP0001828 | CNS0390051 | CNX0330466 | CNR0403260 |
| 02 | 1st strain | CNP0001828 | CNS0390035 | CNX0330450 | CNR0403244 |
|  | 2nd strain | CNP0001828 | CNS0389946 | CNX0330361 | CNR0403155 |
| 03 | 1st strain | CNP0001828 | CNS0390007 | CNX0330422 | CNR0403216 |
|  | 2nd strain | CNP0001828 | CNS0389968 | CNX0330383 | CNR0403177 |
| 04 | 1st strain | CNP0001828 | CNS0389897 | CNX0330312 | CNR0403106 |
|  | 2nd strain | CNP0001828 | CNS0389898 | CNX0330313 | CNR0403107 |
| 05 | 1st strain | CNP0001828 | CNS0389899 | CNX0330314 | CNR0403108 |
|  | 2nd strain | CNP0001828 | CNS0389900 | CNX0330315 | CNR0403109 |
| 06 | 1st strain | CNP0001828 | CNS0389970 | CNX0330385 | CNR0403179 |
|  | 2nd strain | CNP0001828 | CNS0389971 | CNX0330386 | CNR0403180 |
| 07 | 1st strain | CNP0001828 | CNS0389955 | CNX0330370 | CNR0403164 |
|  | 2nd strain | CNP0001828 | CNS0389956 | CNX0330371 | CNR0403165 |
| 08 | 1st strain | CNP0001828 | CNS0389938 | CNX0330353 | CNR0403147 |
|  | 2nd strain | CNP0001828 | CNS0389939 | CNX0330354 | CNR0403148 |
| 09 | 1st strain | CNP0001828 | CNS0389901 | CNX0330316 | CNR0403110 |
|  | 2nd strain | CNP0001828 | CNS0389902 | CNX0330317 | CNR0403111 |
| 10 | 1st strain | CNP0001828 | CNS0389964 | CNX0330379 | CNR0403173 |
|  | 2nd strain | CNP0001828 | CNS0389965 | CNX0330380 | CNR0403174 |
| 11 | 1st strain | CNP0001828 | CNS0390018 | CNX0330433 | CNR0403227 |
|  | 2nd strain | CNP0001828 | CNS0390045 | CNX0330460 | CNR0403254 |
| 12 | 1st strain | CNP0001828 | CNS0390052 | CNX0330467 | CNR0403261 |
|  | 2nd strain | CNP0001828 | CNS0390053 | CNX0330468 | CNR0403262 |
| 13 | 1st strain | CNP0001828 | CNS0389936 | CNX0330351 | CNR0403145 |
|  | 2nd strain | CNP0001828 | CNS0389937 | CNX0330352 | CNR0403146 |
| 14 | 1st strain | CNP0001828 | CNS0389952 | CNX0330367 | CNR0403161 |
|  | 2nd strain | CNP0001828 | CNS0390050 | CNX0330465 | CNR0403259 |
| 15 | 1st strain | CNP0001828 | CNS0389962 | CNX0330377 | CNR0403171 |
|  | 2nd strain | CNP0001828 | CNS0389963 | CNX0330378 | CNR0403172 |
| 16 | 1st strain | CNP0001828 | CNS0390044 | CNX0330459 | CNR0403253 |
|  | 2nd strain | CNP0001828 | CNS0389984 | CNX0330399 | CNR0403193 |
| 17 | 1st strain | CNP0001828 | CNS0389931 | CNX0330346 | CNR0403140 |
|  | 2nd strain | CNP0001828 | CNS0389932 | CNX0330347 | CNR0403141 |
| 18 | 1st strain | CNP0001828 | CNS0389925 | CNX0330340 | CNR0403134 |
|  | 2nd strain | CNP0001828 | CNS0389926 | CNX0330341 | CNR0403135 |
| 19 | 1st strain | CNP0001828 | CNS0390005 | CNX0330420 | CNR0403214 |
|  | 2nd strain | CNP0001828 | CNS0389992 | CNX0330407 | CNR0403201 |
| 20 | 1st strain | CNP0001828 | CNS0390021 | CNX0330436 | CNR0403230 |
|  | 2nd strain | CNP0001828 | CNS0389991 | CNX0330406 | CNR0403200 |
| 21 | 1st strain | CNP0001828 | CNS0389923 | CNX0330338 | CNR0403132 |
|  | 2nd strain | CNP0001828 | CNS0389924 | CNX0330339 | CNR0403133 |
| 22 | 1st strain | CNP0001828 | CNS0389903 | CNX0330318 | CNR0403112 |
|  | 2nd strain | CNP0001828 | CNS0389904 | CNX0330319 | CNR0403113 |
| 23 | 1st strain | CNP0001828 | CNS0389953 | CNX0330368 | CNR0403162 |
|  | 2nd strain | CNP0001828 | CNS0389954 | CNX0330369 | CNR0403163 |
| 24 | 1st strain | CNP0001828 | CNS0390019 | CNX0330434 | CNR0403228 |
|  | 2nd strain | CNP0001828 | CNS0389993 | CNX0330408 | CNR0403202 |
| 25 | 1st strain | CNP0001828 | CNS0390024 | CNX0330439 | CNR0403233 |
|  | 2nd strain | CNP0001828 | CNS0389966 | CNX0330381 | CNR0403175 |
| 26 | 1st strain | CNP0001828 | CNS0390006 | CNX0330421 | CNR0403215 |
|  | 2nd strain | CNP0001828 | CNS0390003 | CNX0330418 | CNR0403212 |
| 27 | 1st strain | CNP0001828 | CNS0389982 | CNX0330397 | CNR0403191 |
|  | 2nd strain | CNP0001828 | CNS0390020 | CNX0330435 | CNR0403229 |
| 28 | 1st strain | CNP0001828 | CNS0390004 | CNX0330419 | CNR0403213 |
|  | 2nd strain | CNP0001828 | CNS0390034 | CNX0330449 | CNR0403243 |
| 29 | 1st strain | CNP0001828 | CNS0389947 | CNX0330362 | CNR0403156 |
|  | 2nd strain | CNP0001828 | CNS0390038 | CNX0330453 | CNR0403247 |
| 30 | 1st strain | CNP0001828 | CNS0390023 | CNX0330438 | CNR0403232 |
|  | 2nd strain | CNP0001828 | CNS0389957 | CNX0330372 | CNR0403166 |
| 31 | 1st strain | CNP0001828 | CNS0390022 | CNX0330437 | CNR0403231 |
|  | 2nd strain | CNP0001828 | CNS0389986 | CNX0330401 | CNR0403195 |
| 32 | 1st strain | CNP0001828 | CNS0389967 | CNX0330382 | CNR0403176 |
|  | 2nd strain | CNP0001828 | CNS0390027 | CNX0330442 | CNR0403236 |
| 33 | 1st strain | CNP0001828 | CNS0389927 | CNX0330342 | CNR0403136 |
|  | 2nd strain | CNP0001828 | CNS0389928 | CNX0330343 | CNR0403137 |
| 34 | 1st strain | CNP0001828 | CNS0390041 | CNX0330456 | CNR0403250 |
|  | 2nd strain | CNP0001828 | CNS0389972 | CNX0330387 | CNR0403181 |
| 35 | 1st strain | CNP0001828 | CNS0389929 | CNX0330344 | CNR0403138 |
|  | 2nd strain | CNP0001828 | CNS0389930 | CNX0330345 | CNR0403139 |
| 36 | 1st strain | CNP0001828 | CNS0390016 | CNX0330431 | CNR0403225 |
|  | 2nd strain | CNP0001828 | CNS0389988 | CNX0330403 | CNR0403197 |
| 37 | 1st strain | CNP0001828 | CNS0389980 | CNX0330395 | CNR0403189 |
|  | 2nd strain | CNP0001828 | CNS0389981 | CNX0330396 | CNR0403190 |
| 38 | 1st strain | CNP0001828 | CNS0389934 | CNX0330349 | CNR0403143 |
|  | 2nd strain | CNP0001828 | CNS0389935 | CNX0330350 | CNR0403144 |
| 39 | 1st strain | CNP0001828 | CNS0390032 | CNX0330447 | CNR0403241 |
|  | 2nd strain | CNP0001828 | CNS0390025 | CNX0330440 | CNR0403234 |
| 40 | 1st strain | CNP0001828 | CNS0389985 | CNX0330400 | CNR0403194 |
|  | 2nd strain | CNP0001828 | CNS0390033 | CNX0330448 | CNR0403242 |
| 41 | 1st strain | CNP0001828 | CNS0390013 | CNX0330428 | CNR0403222 |
|  | 2nd strain | CNP0001828 | CNS0390048 | CNX0330463 | CNR0403257 |
| 42 | 1st strain | CNP0001828 | CNS0390046 | CNX0330461 | CNR0403255 |
|  | 2nd strain | CNP0001828 | CNS0390043 | CNX0330458 | CNR0403252 |
| 43 | 1st strain | CNP0001828 | CNS0390010 | CNX0330425 | CNR0403219 |
|  | 2nd strain | CNP0001828 | CNS0389908 | CNX0330323 | CNR0403117 |
| 44 | 1st strain | CNP0001828 | CNS0389922 | CNX0330337 | CNR0403131 |
|  | 2nd strain | CNP0001828 | CNS0390049 | CNX0330464 | CNR0403258 |
| 45 | 1st strain | CNP0001828 | CNS0389983 | CNX0330398 | CNR0403192 |
|  | 2nd strain | CNP0001828 | CNS0390039 | CNX0330454 | CNR0403248 |
| 46 | 1st strain | CNP0001828 | CNS0390040 | CNX0330455 | CNR0403249 |
|  | 2nd strain | CNP0001828 | CNS0390030 | CNX0330445 | CNR0403239 |
| 47 | 1st strain | CNP0001828 | CNS0390011 | CNX0330426 | CNR0403220 |
|  | 2nd strain | CNP0001828 | CNS0390012 | CNX0330427 | CNR0403221 |
| 48 | 1st strain | CNP0001828 | CNS0390042 | CNX0330457 | CNR0403251 |
|  | 2nd strain | CNP0001828 | CNS0390009 | CNX0330424 | CNR0403218 |
| 49 | 1st strain | CNP0001828 | CNS0390015 | CNX0330430 | CNR0403224 |
|  | 2nd strain | CNP0001828 | CNS0389909 | CNX0330324 | CNR0403118 |
| 50 | 1st strain | CNP0001828 | CNS0389910 | CNX0330325 | CNR0403119 |
|  | 2nd strain | CNP0001828 | CNS0390028 | CNX0330443 | CNR0403237 |
| 51 | 1st strain | CNP0001828 | CNS0389974 | CNX0330389 | CNR0403183 |
|  | 2nd strain | CNP0001828 | CNS0389975 | CNX0330390 | CNR0403184 |
| 52 | 1st strain | CNP0001828 | CNS0390026 | CNX0330441 | CNR0403235 |
|  | 2nd strain | CNP0001828 | CNS0390017 | CNX0330432 | CNR0403226 |
| 53 | 1st strain | CNP0001828 | CNS0389976 | CNX0330391 | CNR0403185 |
|  | 2nd strain | CNP0001828 | CNS0389977 | CNX0330392 | CNR0403186 |
| 54 | 1st strain | CNP0001828 | CNS0390031 | CNX0330446 | CNR0403240 |
|  | 2nd strain | CNP0001828 | CNS0390014 | CNX0330429 | CNR0403223 |
| 55 | 1st strain | CNP0001828 | CNS0389978 | CNX0330393 | CNR0403187 |
|  | 2nd strain | CNP0001828 | CNS0389979 | CNX0330394 | CNR0403188 |
| 56 | 1st strain | CNP0001828 | CNS0389911 | CNX0330326 | CNR0403120 |
|  | 2nd strain | CNP0001828 | CNS0389912 | CNX0330327 | CNR0403121 |
| 57 | 1st strain | CNP0001828 | CNS0389913 | CNX0330328 | CNR0403122 |
|  | 2nd strain | CNP0001828 | CNS0389914 | CNX0330329 | CNR0403123 |
| 58 | 1st strain | CNP0001828 | CNS0389940 | CNX0330355 | CNR0403149 |
|  | 2nd strain | CNP0001828 | CNS0389941 | CNX0330356 | CNR0403150 |
| 59 | 1st strain | CNP0001828 | CNS0389915 | CNX0330330 | CNR0403124 |
|  | 2nd strain | CNP0001828 | CNS0389916 | CNX0330331 | CNR0403125 |
| 60 | 1st strain | CNP0001828 | CNS0389958 | CNX0330373 | CNR0403167 |
|  | 2nd strain | CNP0001828 | CNS0389959 | CNX0330374 | CNR0403168 |
| 61 | 1st strain | CNP0001828 | CNS0389942 | CNX0330357 | CNR0403151 |
|  | 2nd strain | CNP0001828 | CNS0389943 | CNX0330358 | CNR0403152 |
| 62 | 1st strain | CNP0001828 | CNS0389918 | CNX0330333 | CNR0403127 |
|  | 2nd strain | CNP0001828 | CNS0389919 | CNX0330334 | CNR0403128 |
| 63 | 1st strain | CNP0001828 | CNS0389920 | CNX0330335 | CNR0403129 |
|  | 2nd strain | CNP0001828 | CNS0389921 | CNX0330336 | CNR0403130 |
| 64 | 1st strain | CNP0001828 | CNS0389999 | CNX0330414 | CNR0403208 |
|  | 2nd strain | CNP0001828 | CNS0390000 | CNX0330415 | CNR0403209 |
| 65 | 1st strain | CNP0001828 | CNS0389944 | CNX0330359 | CNR0403153 |
|  | 2nd strain | CNP0001828 | CNS0389945 | CNX0330360 | CNR0403154 |
| 66 | 1st strain | CNP0001828 | CNS0389948 | CNX0330363 | CNR0403157 |
|  | 2nd strain | CNP0001828 | CNS0389949 | CNX0330364 | CNR0403158 |
| 67 | 1st strain | CNP0001828 | CNS0389960 | CNX0330375 | CNR0403169 |
|  | 2nd strain | CNP0001828 | CNS0389961 | CNX0330376 | CNR0403170 |
| 68 | 1st strain | CNP0001828 | CNS0390029 | CNX0330444 | CNR0403238 |
|  | 2nd strain | CNP0001828 | CNS0389917 | CNX0330332 | CNR0403126 |

# Supplementary Fuigres

Figure S1 A minimum spanning tree based on pairwise whole genome SNP differences of paired strains.


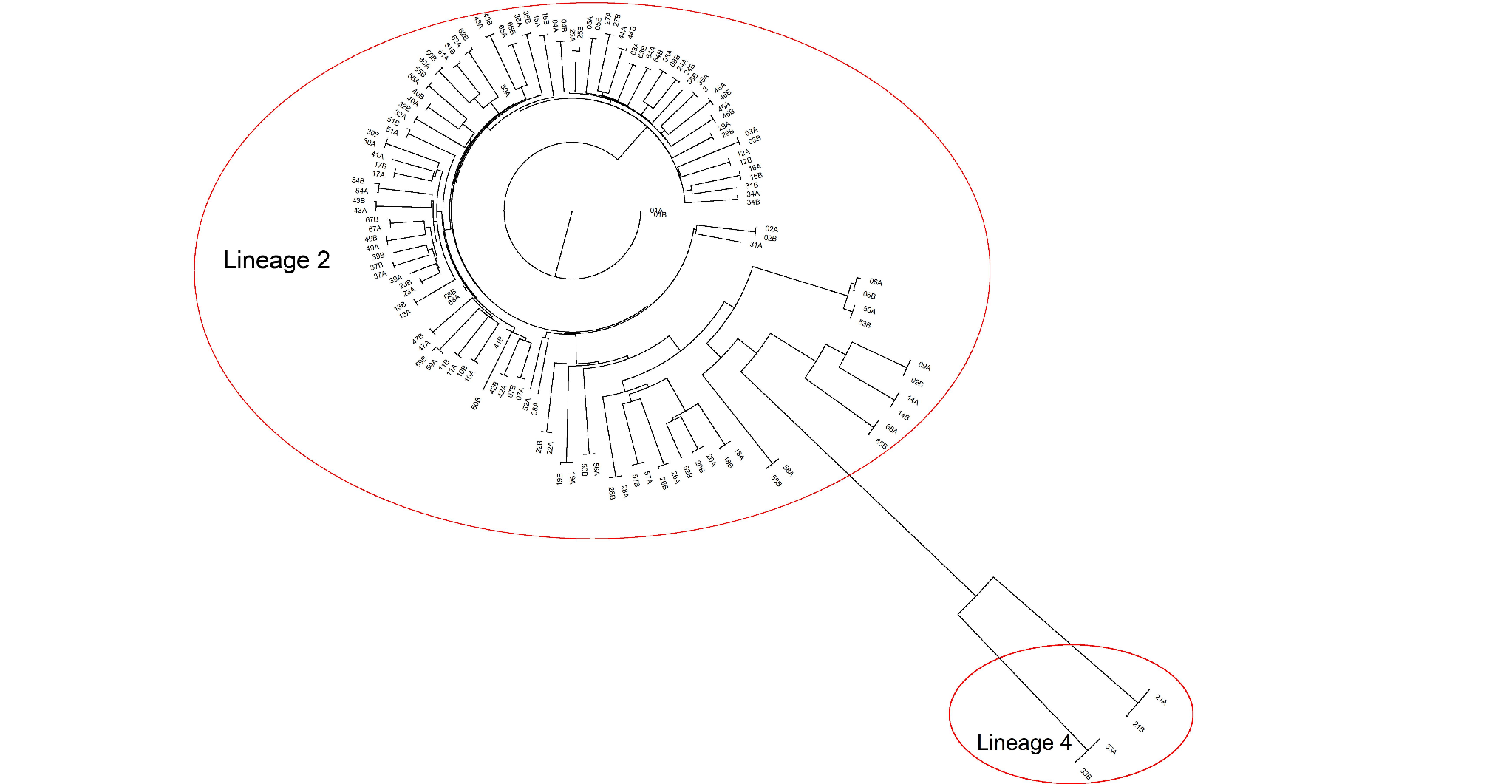

Supplement: Supplementary file 1 [file Table_1.DOCX]
